# Support Effect of Boron Nitride on the First N-H Bond Activation of NH_3_ on Ru Clusters

**DOI:** 10.3390/molecules29020328

**Published:** 2024-01-09

**Authors:** Li Zhao, Huimin Zhuang, Yixuan Zhang, Lishuang Ma, Yanyan Xi, Xufeng Lin

**Affiliations:** 1 College of Chemistry and Chemical Engineering, China University of Petroleum (East China), Qingdao 266580, China; zhaoli1175@163.com (L.Z.); m15698073639@163.com (Y.Z.); hxmals@upc.edu.cn (L.M.); xiyy@upc.edu.cn (Y.X.); 2 Shandong Yellow Sea Institute of Science and Technology Innovation, Rizhao 276808, China; 13156382598@163.com; 3 Advanced Chemical Engineering and Energy Materials Research Center, China University of Petroleum (East China), Qingdao 266580, China; 4 State Key Laboratory of Heavy Oil Processing, China University of Petroleum (East China), Qingdao 266580, China

**Keywords:** N-H bond activation of ammonia, support effect, boron nitride, ruthenium, density functional theory calculation

## Abstract

Support effect is an important issue in heterogeneous catalysis, while the explicit role of a catalytic support is often unclear for catalytic reactions. A systematic density functional theory computational study is reported in this paper to elucidate the effect of a model boron nitride (BN) support on the first N-H bond activation step of NH_3_ on Ru_n_ (*n* = 1, 2, 3) metal clusters. Geometry optimizations and energy calculations were carried out using density functional theory (DFT) calculation for intermediates and transition states from the starting materials undergoing the N-H activation process. The primary findings are summarized as follows. The involvement of the model BN support does not significantly alter the equilibrium structure of intermediates and transition states in the most favorable pathway (MFP). Moreover, the involvement of BN support decreases the free energy of activation, Δ*G*^≠^, thus improving the reaction rate constant. This improvement is more obvious at high temperatures like 673 K than low temperatures like 298 K. The BN support effect leading to the Δ*G*^≠^ decrease is most significant for the single Ru atom case among all three cases studied. Finally, the involvement of the model BN may change the spin transition behavior of the reaction system during the N-H bond activation process. All these findings provide a deeper insight into the support effect on the N-H bond activation of NH_3_ for the supported Ru catalyst in particular and for supported transition metal catalysts in general.

## 1. Introduction

Bockris introduced the concept of a “hydrogen (H_2_) economy” envisioning an energy transition founded on the utilization of H_2_ as a vector for the generation of clean and environmentally sustainable energy [1]. Over recent decades, the production of H_2_ from various sources, its transport and storage, and finally its use have been extensively investigated [2]. H_2_ has assumed a prominent role in the energy sector, finding applications in stationary power generation, transportation, and as an energy vector for storing surplus electrical energy generated during off-peak periods [3]. However, one of the challenges in H_2_ technology today is storage and transportation. Due to the problems of physical storage methods, chemical storage methods based on using another easily transportable hydrogen-containing compound, which in turn produces H_2_ by chemical reaction, may be more favored. Ammonia (NH_3_) is currently one of the most promising H_2_ carriers. It can form a liquid at low pressure at ambient temperature, it is easy to transport and store, and its industrial synthesis is mature. If the cost-effective production of H_2_ through NH_3_ decomposition can be achieved, it is anticipated that H_2_ storage and transportation via NH_3_ will exhibit significant technical and economic competitiveness.

In recent years, NH_3_ decomposition to produce H_2_ has received more and more attention by people in fundamental research and industrial applications [4,5,6,7,8,9,10,11,12]. Because of the inertia of NH_3_, its activation and conversion to N_2_ and H_2_ has to involve catalysis. The activation of the N-H bond is one of the key steps as well as the first step in the catalytic conversion of NH_3_. The activation mechanism of the N-H bond is undoubtedly important for understanding the existing NH_3_ catalytic conversion processes and developing new NH_3_ catalytic conversion systems. Supported nickel (Ni) and supported ruthenium (Ru) catalysts are the most commonly used catalysts in fundamental research and in pilot plants [4,5,8,9,10,11,12]. Many researchers have studied the reaction kinetics or/and reaction mechanisms of NH_3_ decomposition catalyzed by Ru- or Ni-based catalysts [12,13]. Yue et al. [14] summarized several ways of the N-H bond activation on metal catalysts. They also proposed that the difficulty of N-H bond activation of NH_3_ is due to the relatively large N-H bond energy and the relative activity of the lone pair of electrons on the N atom. However, these mechanistic point of views focused on reactions of organic synthesis. Piers et al. [15] reported the N-H bond activation of NH_3_ via reaction with low-valence molybdenum complexes of a diborate pentadentate ligand system.

**Scheme 1:**
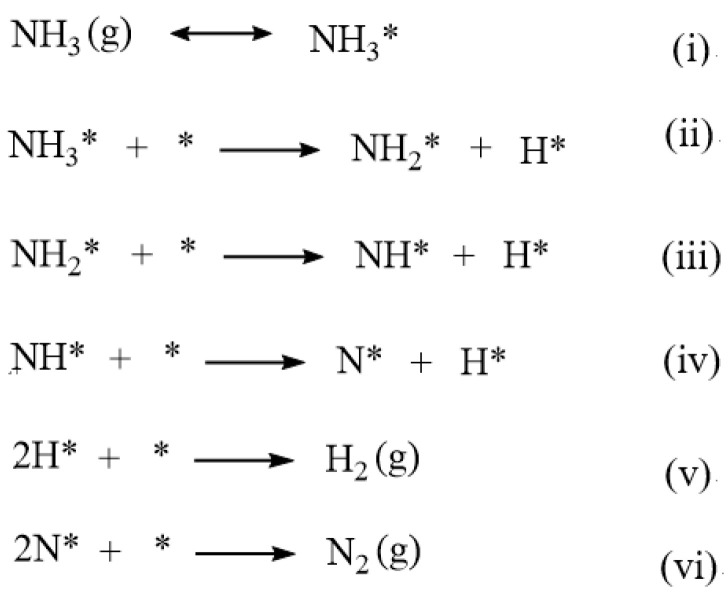
A reaction mechanism described by Sun in Ref. [24] and the references therein.

In a broader scope of the NH_3_ decomposition mechanism on transition metal catalysts, most of the research has been carried out with a plane surface model like Pd(111) [16], Ni(111), Co(111), Fe(110) [17], Cu(111) [18], Cu(100) [19], and WC(0001) [20]. For example, Jiang et al. [16] reported that NH is the most abundant intermediate on the Pd (111) surface and the dehydrogenation of NH_3_ is the rate-determining step in the overall reaction. However, the kinetic analysis and mechanistic studies of NH_3_ decomposition on Ni and Ru catalysts have not yet led to being clearly studied in detail [21]. Especially, the activation of N-H bonds on the metal atom/clusters is still not well understood, at the level of elementary steps and at the molecular level [4,22,23]. For example, a well-recognized reaction pathway of NH_3_ decomposition can be expressed as described in Scheme 1 [24].

Scheme 1, as described by Sun in Ref. [24] and the references therein, outlines a reaction mechanism in which the symbol “*” designates the reactive site responsible for NH_3_ decomposition. The initial two steps within Scheme 1 correspond to the initial activation of the first N-H bond in NH_3_ on a specific catalytic site. However, the comprehensive understanding of the reaction mechanism presented in Scheme 1, particularly within these initial two steps, remains elusive. This holds especially true for critical information regarding the nature of the catalytic site represented by “*”. Several unresolved questions come to light, particularly concerning these initial two steps in Scheme 1. For example, firstly, it is not clear whether the two “*” notations in the first two steps correspond to the same metal site or two different metal sites. It should be noted that the answer may be different when dealing with a single metal atom and with metal clusters. It is worth noting that the answer to this question may differ when considering a single metal atom as opposed to metal clusters. Secondly, there is a lack of understanding regarding how the incorporation of a catalytic support into the metal site alters the reaction behaviors. These questions are certainly important since catalytically active metal components are always resided on a frequently used support, like Al_2_O_3_, carbon nanotube, graphene, and boron nitride (BN). Support effect is an important topic in heterogeneous catalysis, in particular for Ru-catalyzed hydrogen utilization processes [25], and for NH_3_ decomposition to obtain high-purity hydrogen [26].

To gain a clearer comprehension of the impact of catalytic support and metal cluster size on N-H bond activation behavior in metal atom/clusters, we present a DFT study of N-H bond activation of NH_3_ on both of the unsupported and supported Ru atom/clusters in this paper. This paper focuses on the structural, energetic, and spin multiplicity changes during the N-H bond activation process without and with the model hexagonal BN support. BN, like its C analogue, can exist in various forms like hexagonal sheets, nanotubes, and nanobowls, resulting in various interesting properties and being useful in the field of catalysis [27,28,29]. We found that the model support can change the structure of intermediates and transition states and decrease the reaction energy barrier. The results presented in this work offer valuable qualitative insights, contributing to a deeper understanding of the impact of catalytic support and metal cluster size on the activation of N-H bonds and other types of saturated bonds in a broader context.

## 2. Results and Discussion

### 2.1. Adsorption Energy of Ru_n_ Atom/Clusters on the BN Support

Before studying the support effect of BN on the N-H bond activation process, the stability of Ru_n_ clusters on the BN support should be first examined. In general, a transition metal center can have more than one accessible spin state, which can be close in energy to each other. In particular, as is well known, an isolated Ru atom has a ground state in its quintet (spin multiplicity, *S* = 5, denoted as ^5^Ru) state since its ground state electron configuration is [Kr]4d^7^5s^1^. However, when an Ru atom interacts with other entities like a second Ru atom to form an Ru_2_ cluster, or an NH_3_ molecule for further reaction, it is possible to change its ground state spin multiplicity. The change in spin multiplicity is always not possible to increase, since the incorporation of another entity into an Ru atom lowers its symmetry. Similarly, when an Ru_n_ cluster interacts with the BN support, the ground spin multiplicity may also change in principle.

Therefore, in order to calculate the adsorption energy of the Ru clusters on BN support, one needs to compare the energy of the Ru_n_ clusters for both of the unsupported and supported cases with different spin multiplicities. All the species notations shown in the first column in Table 1 correspond to their ground states after a similar energy calculation process as the case of Ru_1_-BN. For instance, ^5^Ru_1_-BN is considered as the ground state since its energy is lower than ^1^Ru_1_-BN, ^3^Ru_1_-BN, and ^7^Ru_1_-BN. ^9^Ru_3_ is considered as the ground state since its energy is lower than ^1^Ru_3_, ^3^Ru_3_, ^5^Ru_3_, ^7^Ru_3_, and ^11^Ru_3_. In addition, the model BN sheet has a singlet ground state since a triplet BN sheet has a much higher energy.

**Figure 1:**
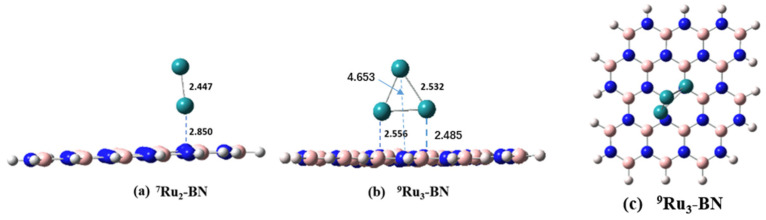
Side view of the model BN supported ^7^Ru_2_ (**a**, ^7^Ru_2_-BN) and ^9^Ru_3_ (**b**, ^9^Ru_3_-BN) clusters, and top-down view of ^9^Ru_3_-BN (**c**). Key distances are indicated in Å.

Figure 1 shows the optimized geometries of ^7^Ru_2_-BN and ^9^Ru_3_-BN. Interestingly the two Ru atoms tend to stand perpendicular rather than lie parallel to the BN plane in ^7^Ru_2_-BN. The triangular plane formed from the three Ru atoms also tends to stand perpendicular to the BN plane. Various initially designed structures were considered, such as placing Ru atom(s) at the center of a BN hexagonal ring, a B-N bridge site, as well as positions directly attached to a B or N atoms. These initial structures were subjected to geometry optimization to identify the most stable configuration. Notably, after optimization, the lowest-energy structure for all initially designed configurations featured Ru atoms positioned closer to B atoms, as shown in Figure 1. Table 1 shows that the adsorption energies (*E*_ad_ < 0) of these Ru clusters on the BN sheet are moderately high, making the adsorption process feasible (*G*_ad_ < 0).

### 2.2. Support Effect on the First N-H Bond Activation Process of NH_3_ on One Ru Atom

#### 2.2.1. Structure

**Figure 2:**
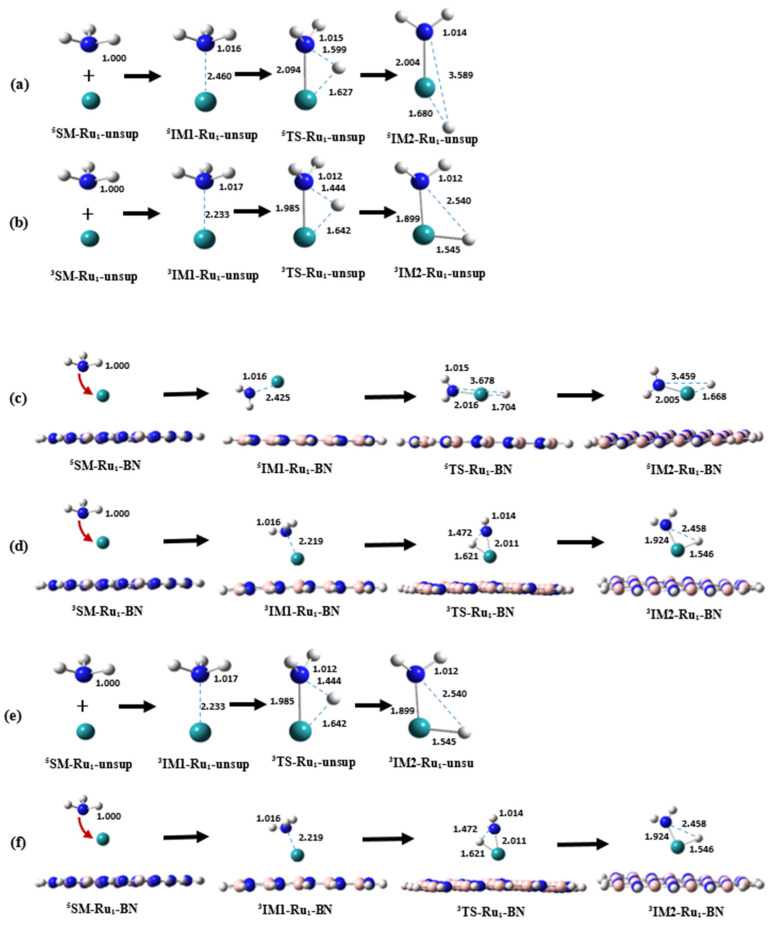
Shown are the optimized geometries of the key species involved in the first N-H bond activation of NH_3_ on unsupported Ru_1_ ((R1) with suffix of **-Ru_1_-unsup**) and the model BN-supported Ru_1_ ((R4) with suffix of **-Ru_1_-BN**). Panels (**a**,**b**) illustrate the unsupported cases on the quintet and triplet PES, respectively, while (**c**,**d**) demonstrate the supported cases on the quintet and triplet PES. Panel (**e**) shows the cases of (R1) with the most favorable pathway (MFP), and (**f**) for (R4). All distances are indicated in Å.

As mentioned above, since Ru_1_ has a quintet ground state, in this work the reaction behavior of the first N-H bond activation of NH_3_ on a ^5^Ru atom was investigated in the beginning. Figure 2a presents the optimized geometries of the species participating in the NH_3_ and ^5^Ru reaction ((R1) as defined in Section 3). The reaction commences with the isolated NH_3_ molecule and a ^5^Ru atom, serving as the starting materials (designated as **^5^SM-Ru_1_-unsup**). As the reaction progresses, the NH_3_ molecule approaches the ^5^Ru atom, resulting in the formation of the first energy minimum structure, denoted as **^5^IM1-Ru_1_-unsup**, with an N…Ru distance of 2.460 Å. Concurrently, the N-H bond containing the detaching H atom (referred to as H_a_ hereafter) experiences a slight increase in length. Subsequently, **^5^IM1-Ru_1_-unsup** evolves into another intermediate, **^5^IM2-Ru1-unsup**, via a transition state structure denoted as **^5^TS-Ru_1_-unsup**. During this transition, H_a_ detaches from the N atom, moving closer to the Ru atom, while the N atom further approaches Ru. These structural alterations are evident in Figure 2a, where, for instance, the N…H_a_ distance increases from 1.016 Å in **^5^IM1-Ru_1_-unsup** to 1.599 Å in **^5^TS-Ru_1_-unsup** and then to 3.589 Å in **^5^IM2-Ru_1_-unsup**. In the **^5^IM2-Ru_1_-unsup** intermediate, the Ru-H_a_ bond is fully formed, as indicated by its length of 1.680 Å.

Later, the PESs in the singlet, triplet, and heptet states (*S* = 1, 3, and 7, respectively) were also investigated in this work similar to the quintet PES case. Since the energies of the **SM**, **IM1**, **TS**, and **IM2** species on the singlet and heptet PESs are significantly higher than the corresponding species on the triplet and quintet PESs, the results related to the singlet and heptet PESs will not be reported in this paper. The geometrical characters of the key species on the triplet PES (Figure 2b) are rather similar to the ones on the quintet PES. The primary differences are in that the N…Ru and the N…H_a_ distances are often shorter in the triplet species than that in the quintet species for (R1). For example, the N…Ru distance is 1.985 Å in **^3^TS-Ru_1_-unsup** compared to 2.094 Å in **^5^TS-Ru_1_-unsup**, and the N…H_a_ distance is 1.444 Å in **^3^TS-Ru_1_-unsup** compared to 1.599 Å in **^5^TS-Ru_1_-unsup**.

As described in Section 2.1, when an isolated Ru atom resides on a model BN surface to form Ru_1_-BN, quintet is still in the ground spin state compared to the singlet, triplet, and heptet states. Similar to the unsupported case of (R1) described above, the results related to the singlet and heptet PESs will also not be reported for the supported case of (R4). Figure 2c,d show that, except the incorporation of the BN support, the geometries of the Ru…NH_3_ part are somewhat similar to the case of unsupported reaction system, with the main difference in that the N…H_a_ distance in the TS for the supported case is slightly longer than that for the unsupported case. For example, the distance between the detaching atom of the H_a_ and N atom in NH_3_ in ^3^**TS-Ru_1_-unsup** is 1.444 Å (Figure 1b), which is 1.472 Å in ^3^**TS-Ru_1_-BN** (Figure 2d) when the BN support is added to the reaction system. In addition, the distance between H_a_ and Ru atoms becomes shorter after adding the model BN support.

#### 2.2.2. Energy Profiles

**Figure 3:**
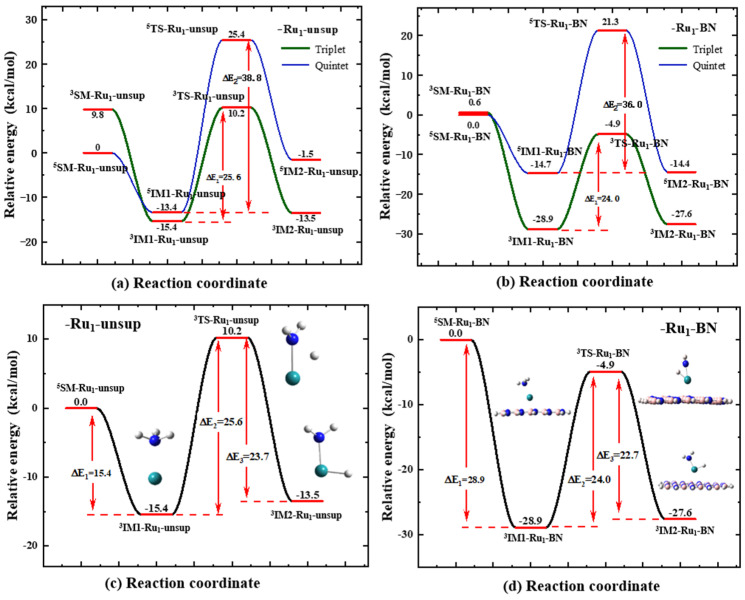
Shown is the relative energy profiles for the first N-H bond activation of NH_3_ on one Ru atom for the unsupported ((R1) with suffix of **-Ru_1_-unsup**) and supported ((R4) with suffix of **-Ru_1_-BN**). The relative energy profiles on the triplet (green) and quintet (blue) PESs are included in panel (**a**) for (R1) and in panel (**b**) for (R4). on **Ru_1_-unsup** (**a**) and **Ru_1_-BN** (**b**). The relative energy profiles of the most favorable pathway (MPF) are shown in panel (**c**) for (R1) and in panel (**d**) for (R4).

Figure 3a,b show the relative energy profiles for (R1) and (R4) undergoing on the quintet and triplet PESs, respectively. For (R1), ^3^**SM-Ru_1_-unsup** is higher in energy than **^5^SM-Ru_1_-unsup**, which is not surprising since an Ru atom has a quintet ground state. However, it is noteworthy that the intermediate **IM1** has a triplet ground state rather than quintet. In another word, the ground spin state of Ru is changed during the course of an NH_3_ molecule approaching an Ru atom. More importantly, both the transition state **TS** and intermediate **IM2** display lower energy on the triplet PES compared to the quintet PES. The activation energy of (R1), defined as the energy difference between **TS-Ru_1_-unsup** and **IM1-Ru_1_-unsup**, is also lower on the triplet PES (25.6 kcal/mol) than on the quintet PES (38.8 kcal/mol).

**Figure 4:**
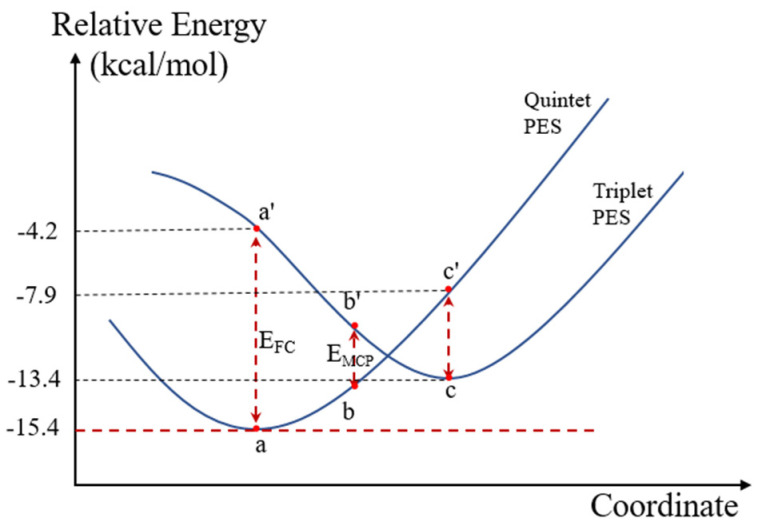
Shown is a simple schematic illustration for the landscape of the energy surface near the energy minimum of triplet and quintet **IM1-Ru_1_-unsup** in (R1). Point a, the energy minimum on the triplet PES, represents the energy of optimized **^3^IM1-Ru_1_-unsup**. Point a′ represents the energy of **IM1-Ru_1_-unsup** in quintet state while having the same geometry as point a. The energy difference between a and a′ is the Frank–Condon excitation energy (E_FC_) at point a. Point b represents the geometry and the energy of **^3^IM1-Ru_1_-unsup** where the spin transition occurs with the largest probability. The energy difference between b and b′ represents the minimum energy at cross point (MECP). Point c is the energy of optimized **^5^IM1-Ru_1_-unsup**. Point c′ represents the energy of **IM1-Ru_1_-unsup** in triplet state while having the same geometry as point c. The energy difference between c and c′ is the E_FC_ at point c, with the geometry of optimized ^5^**IM1-Ru_1_-unsup**.

Examining the energy profiles presented in Figure 3a reveals that **IM1** proceeds much easier to **TS** on the triplet PES than on the quintet PES for (R1) (**^5^IM1-Ru_1_-unusp → ^5^TS-Ru_1_-unusp** vs. **^3^IM1-Ru_1_-unusp → ^3^TS-Ru_1_-unusp**). However, considering that ^5^**SM**-**Ru_1_-unsup** is much more stable than ^3^**SM**-**Ru_1_-unsup** in energy, in this time it is still insufficient to verify the following hypothesis, named as Hypothesis A, i.e., (R1) operates on the triplet PES. On the basis of energetic data, Hypothesis A is valid if Hypothesis B is valid, that is, **^5^IM1-Ru_1_-unsup** can undergo spin transition to ^3^**IM1**-**Ru_1_-unsup** with a low spin transition energy (lower than **^5^IM1-Ru_1_-unsup** going to **^5^TS-Ru_1_-unsup**). An exact calculation of such spin transition energy, belonging to a “spin-forbidden” process problem [30,31], can be achieved by the “minimum energy at crossing point (MECP)” method. Although in this work we did not calculate the MECP value for the **^5^IM1-Ru_1_-unsup** species, through two single-point energy calculations (the results are shown in Figure 4; see more explanation in its figure caption as well) and logical reasoning, Hypothesis B can be verified. Since the energy difference of b → b′ is the MECP, which is certainly smaller than a → a′ or c → c′ (E_b-b′_ < E_a-a′_, E_b-b′_ < E_c-c′_). From Figure 4, the energy differences of points a → a′ and c → c′ are 19.6 and 5.5 kcal/mol, respectively, and thus the energy difference of b → b′, or MECP, is lower than 5.5 kcal/mol. This comparison consequently verifies that **^5^IM1-Ru_1_-unsup** (actually the same point as c) going to **^3^IM1-Ru_1_-unsup** (requiring less than 5.5 kcal/mol) is much easier than it going to **^5^TS-Ru_1_-unsup** (requiring 31.5 kcal/mol, see Figure 2a), that is, Hypothesis B is verified. Therefore, Hypothesis A is also verified.

The verification of Hypothesis A mentioned above shows we can use the concept of the most favorable pathway (MFP) to describe the reaction behavior of (R1). In the MFP energy profile, all key species are considered only with their ground state spin multiplicity. Figure 3c shows the energy profile of the MFP for (R1) deduced from Figure 3a, and correspondingly, the optimized geometries of the key species involved in the MFP for (R1) can be seen in Figure 2e.

In this work we have performed a similar verification process for all other reactions of (R2)~(R5). All of the six reactions in this work have an MFP. Hereafter in this paper, the structures and energy profiles are only reported for the MFPs, instead of presenting the results about all of the spin states.

Similar to the unsupported case of (R1), Figure 3d shows the energy profile of the MFP for the BN-supported case of (R4), which is deduced from the two profiles in Figure 3b, and correspondingly, the optimized geometries of the key species involved in the MFP for (R4) shown in Figure 2f. A comparison between the results in Figure 3c,d reveals that the intermediates of **IM1** and **IM2** are obviously stabilized by ~14 kcal/mol when the BN support is involved. **TS** is lowered by ~15 kcal/mol, leading to the reaction energy barrier, is lowered from 25.6 kcal/mol for the **Ru_1_-unsup** case to 24.0 kcal/mol for the **Ru_1_-BN** case. The reaction barrier of (R4), i.e., the energy difference between **^3^IM1-Ru_1_-BN** and **^3^TS-Ru_1_-unsup**, is 24.0 kcal/mol, which is rather consistent with 1.066 eV (24.6 kcal/mol) for CNT-supported Ru_1_, as reported by Zhou et al. [32]. The results in this work and in Ref. [32] show that Ru can be more effective than Pd for N-H bond activation, since the N-H bond activation barrier is 39.4 kcal/mol on Pd(111) [16].

#### 2.2.3. Effect of the BN Model Size

**Figure 5:**
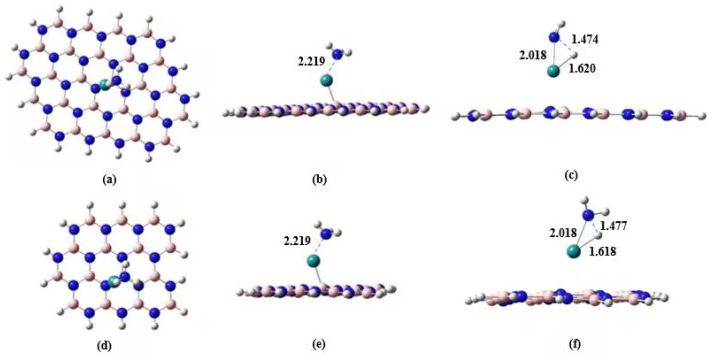
Shown are the optimized geometries of **IM1** and **TS** involved in the first N-H bond activation of NH_3_ on the varied model BN-supported Ru_1_ ((R4) with different sizes of BN model sheet). (**a**) ^3^**IM1-Ru_1_-BN** with a B_26_N_26_H_18_ model in vertical view, (**b**) ^3^**IM1-Ru_1_-BN** with a B_26_N_26_H_18_ model in side view, (**c**) ^3^**TS-Ru_1_-BN** with a B_26_N_26_H_18_ model in side view, (**d**) ^3^**IM1-Ru_1_-BN** with a B_15_N_15_H_14_ model in vertical view, (**e**) ^3^**IM1-Ru_1_-BN** with a B_15_N_15_H_14_ model in side view, and (**f**) ^3^**TS-Ru_1_-BN** with a B_15_N_15_H_14_ model in side view.

Figure 5 show the optimized geometries of **IM1** and **TS** involved in the first N-H bond activation of NH_3_ on the varied model BN-supported Ru_1_ ((R4) with different sizes of BN model sheet). To examine the rationality of the B_19_N_19_H_16_ sheet as a representative model BN sheet, we examined how the reaction barrier of (R4), i.e., energy difference of **^3^IM1-Ru_1_-BN → ^3^TS-Ru_1_-BN**, changed with expanding and reducing the B_19_N_19_H_16_ sheet model. On one hand, we enlarged it to create a B_26_N_26_H_18_ sheet model, and on the other, we reduced it to a B_15_N_15_H_14_ sheet model. These three distinct sheet models were employed as BN support for structural optimization and energy calculations of the intermediates and transition states involved in the N-H bond activation process. The structural characteristics of **IM1** and **TS** derived from these three sheet models exhibited remarkable similarity, as depicted in Figure 2f. Furthermore, the energy results displayed a high degree of consistency. The reaction barrier obtained for the three BN sheet models used as supports are 24.3, 24.0, and 24.3 kcal/mol for B_26_N_26_H_18_, B_19_N_19_H_16_, and B_15_N_15_H_14_, respectively.

These calculations unequivocally demonstrate that the BN models used in this work possess size consistency, thus validating the rationale behind utilizing the B_19_N_19_H_16_ sheet model for BN support calculations. Considering the computational complexity of this work, we ultimately opted for the B_19_N_19_H_16_ sheet model as the BN support for the following calculations. 

### 2.3. Support Effect on the First N-H Bond Activation Process of NH_3_ on an Ru_2_ Cluster

**Figure 6:**
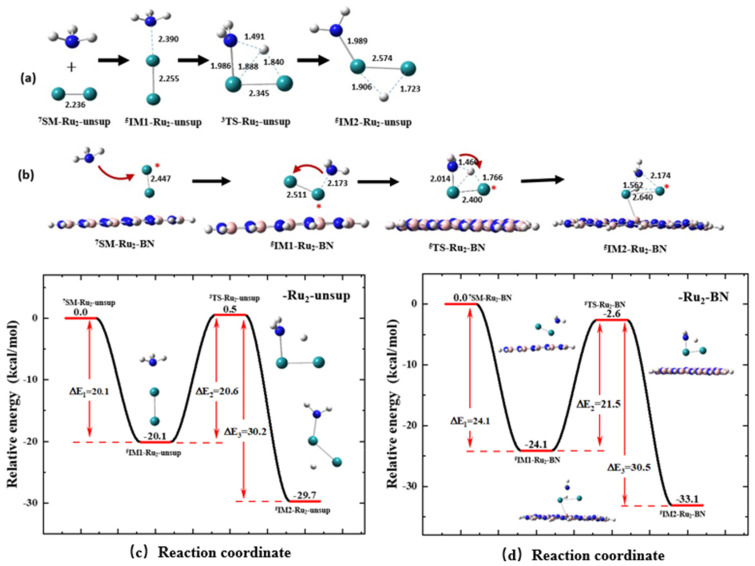
Shown are the optimized geometries of the key species involved in the first N-H bond activation of NH_3_ on unsupported Ru_2_ ((R2) with suffix of **–Ru_2_-unsup**) and the model BN-supported Ru_2_ ((R5) with suffix of **–Ru_2_-BN**) cluster and their relative energy profiles. Panel (**a**) is for (R2) with the most favorable pathway (MFP), and (**b**) for (R5) with the most favorable pathway (MFP). The key distances are indicated in Å. The two Ru atoms are named as Ru-a (indicated with *) and Ru-b in this part for more convenient elaboration. Panels (**c**,**d**) are the relative energy profiles for the first N-H bond activation of NH_3_ on an Ru_2_-unsup (R2) and on an Ru_2_-BN cluster (R5) with the most favorable pathway (MFP).

Similar to the results about Ru_1_ shown in Section 2.2, for all key species involved in the NH_3_ activation process on an unsupported (R2) and supported (R5) Ru_2_ cluster, the geometry optimization and energy calculation were performed with their spin multiplicities of 1, 3, 5, 7, and 9. A species having a quintet (*S* = 5) or heptet (*S* = 7) state is much more stable than it having a singlet, triplet, or nonet state. An unsupported Ru_2_ cluster has a heptet ground state, i.e., ^7^Ru_2_. Figure 6a shows the optimized geometries of the key species involved in the reaction between NH_3_ and an unsupported ^7^Ru_2_ cluster (R2) through the MFP. By comparing between the structures shown in Figure 2e and Figure 6a, it is interesting to identify that, on an Ru_2_ cluster, when the N atom approaches one Ru atom, the detaching H atom, H_a_, approaches the two Ru atoms at the same time during the N-H bond activation process. Finally, the NH_2_ fragment is attached to one Ru atom, and H_a_ is attached to two Ru atoms to form a trigonal H…Ru…Ru structure in ^7^**IM2-Ru_2_-unsup**.

From Figure 6b, it can be seen that the involvement of support significantly changes the stable structure of **IM1** and **IM2**. The incorporation of the support makes ^5^**IM1-Ru_2_-BN** stabilized in a structure that is closer to the transition state, ^5^**TS-Ru_2_-BN**. The BN support also changes the position of H_a_ in ^7^**IM2- Ru_2_-unsup**, forming a structure with the NH_2_ fragment and H_a_ being at the same side, and H_a_ is closer to Ru-a than to Ru-b, as shown in Figure 6b.

Figure 6c shows the energy profile for activation process with the MFP on the **Ru_2_-unsup** cluster (R2). The **SM** of this reaction has a heptet ground state, and as the reaction proceeds, the energy of the reaction system is decreased by 20.1 kcal/mol to reach the first energy minimum, ^5^**IM1-Ru_2_-unsup**. With the low-energy spin transition, the barrier required for ^5^**IM1-Ru_2_-unsup** to proceed to the most favorable TS, ^3^**TS-Ru_2_-unsup,** is 20.6 kcal/mol. Finally, with another spin transition, the energy decreases by 30.2 kcal/mol to reach the second energy minimum of **^5^IM2-Ru_2_-unsup**. Figure 6d shows the energy profile for an activation process with the MFP on the **Ru_2_-BN** cluster (R5). A comparison between the last two panels in Figure 6 shows that the involvement of the BN support leads to a slight increase of 1.0 kcal/mol in the reaction energy barrier. The reaction energy of the elementary step decreases by 3.4 kcal/mol. The reaction barrier of (R5), i.e., the energy difference between **^5^IM1-Ru_2_-BN** and **^5^TS-Ru_2_-BN** is 21.5 kcal/mol, which is also consistent with 0.830 eV (19.1 kcal/mol) for CNT-supported Ru_2_, as reported by Zhou et al. [33].

### 2.4. Support Effect on the First N-H Bond Activation Process of NH_3_ on an Ru_3_ Cluster

**Figure 7:**
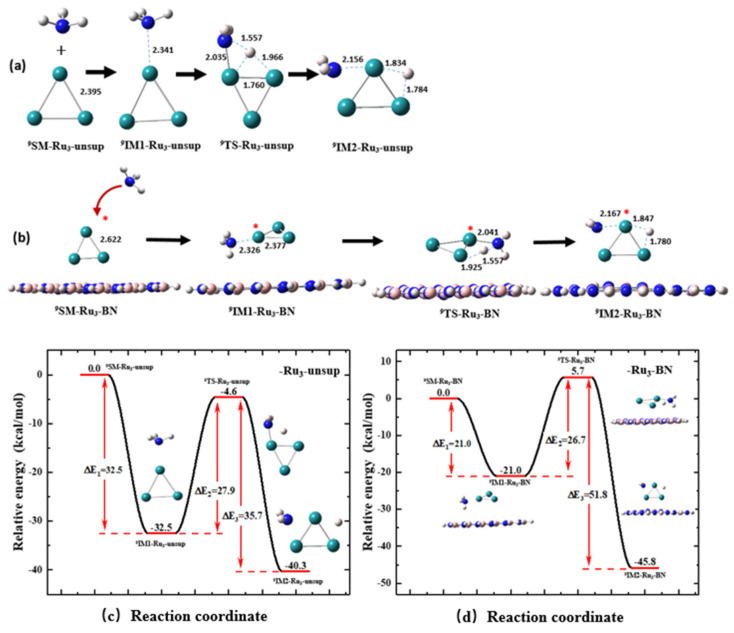
Shown are the optimized geometries of the key species involved in the first N-H bond activation of NH_3_ on unsupported Ru_3_ ((R3) with suffix of **–Ru_3_-unsup**) and the model BN-supported Ru_3_ ((R6) with suffix of **–Ru_3_-BN**) cluster and their relative energy profiles. Panel (**a**) is for (R3) with the most favorable pathway (MFP), and (**b**) for (R6) with the MFP. The Ru atom indicated with “*” is close to the N atom in NH_3_. Panels (**c**,**d**) are the relative energy profiles for the first N-H bond activation of NH_3_ on an Ru_3_-unsup (R3) and on an Ru_3_-BN cluster (R6) with the most favorable pathway (MFP).

Similar to the results about Ru_1_ and Ru_2_ shown in Section 2.2 and Section 2.3, respectively, for all key species involved in the NH_3_ activation process on an unsupported (R3) and supported (R6) Ru_3_ cluster, the geometry optimization and energy calculation were performed with their spin multiplicities of 1, 3, 5, 7, 9, and 11. A species having a nonet (*S* = 9) state is significantly more stable than one with another spin state. An unsupported Ru_3_ cluster has a nonet ground state, i.e., ^9^Ru_3_. Figure 7a illustrates the optimized geometries of the key species involved in the reaction between NH_3_ and an unsupported ^9^Ru_3_ cluster through the MFP (R3). Comparing the structures shown in Figure 2e, Figure 6a and Figure 7a, it can be seen that on an Ru_3_ cluster, similar to the cases of Ru_1_ and Ru_2_, when the N atom approaches one Ru atom, the detaching H atom, H_a_ approaches one of the Ru atoms at the same time during the N-H bond activation process. Finally, the NH_2_ fragment is attached to one Ru atom, and H_a_ is attached to two Ru atoms to form a trigonal H…Ru…Ru structure in ^9^**IM2-Ru_3_-BN**, similar as the Ru_2_ case.

Figure 7a,b shows that the involvement of the BN support changes the structure of **TS** by changing the position of H_a_ atom, from being roughly at the same plane with three Ru atoms to being out of the plane. The involvement of BN also slightly changes the structure of **IM2**.

Figure 7c shows the energy profile for the N-H bond activation process through the MFP on the **Ru_3_-unsup** cluster (R3). The **SM** of this reaction has a nonet ground state, **^9^SM-Ru_3_-unsup**, and as the reaction proceeds, the energy of the reaction system is decreased by 32.5 kcal/mol to reach the first energy minimum, **^9^IM1-Ru_3_-unsup**. As the reaction continues, the energy barrier required for **^9^IM1-Ru_3_-unsup** to proceed to **^9^TS-Ru_3_-unsup** is 27.9 kcal/mol. Finally, the energy decreases by 35.7 kcal/mol to reach the second energy minimum, **^9^IM2-Ru_3_-unsup**. Figure 7d shows the energy profile for the activation process with MFP on the **Ru_3_-BN** cluster (R6). From the last two panels in Figure 7, the involvement of the BN support leads to a slight decrease of 0.9 kcal/mol in the reaction energy barrier. The reaction energy of the elementary step decreases by −5.5 kcal/mol.

### 2.5. Further Discussion on the Support Effect for the First N-H Bond Activation of NH_3_ on Ru_n_ (n = 1, 2, 3) Clusters

**Figure 8:**
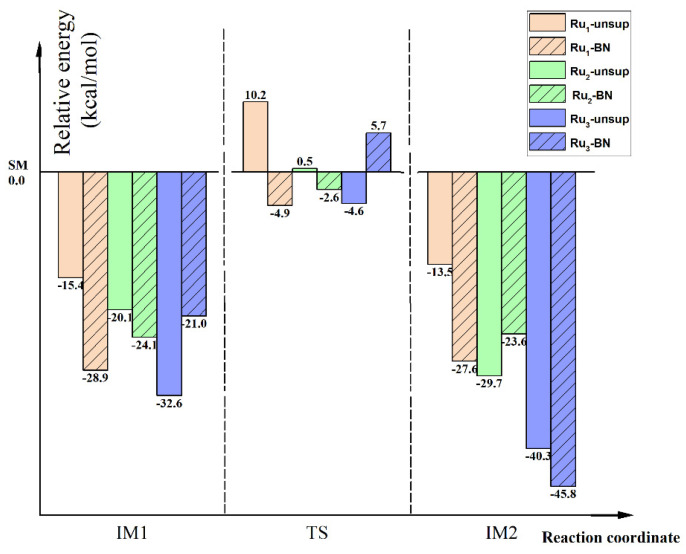
Relative energy of all key species involved in the MFP of the first N-H bond activation of NH_3_ on unsupported and BN-supported Ru_n_ (*n* = 1, 2, 3) clusters, as expressed by (R1)~(R6).

In order to better understand the role of the BN support with different Ru cluster sizes, the relative energy and relative free energy at 298.15 K and 673.15 K of all the key species involved in the MFP of the six reactions studied in this work are collected in Figure 8, with their corresponding **SM**s being chosen as the energetic reference. Based on Figure 8, the support effect on the thermodynamic aspect, kinetic aspect, and the size effect aspect can be more clearly seen.

#### 2.5.1. Thermodynamic Aspect of Support Effect

The incorporation of BN favors the stability of **IM1** and **TS** for the Ru_1_ and Ru_2_ clusters, and disfavors the stability of **IM1** and **TS** for the Ru_3_ cluster. Compared to the Ru_1_ case, the stability of these two states is less favored by BN support for the Ru_2_ case. From the reaction energy point of view, formation of **IM2** from **SM** is favored by the incorporation of BN for all Ru_n_ clusters.

#### 2.5.2. Kinetic Aspect of Support Effect

Based on the transition state theory, the relationship between the rate constant (*k*) and the molar Gibbs free energy of activation (Δ*G*^≠^) is expressed as [34]

*k =* (*k_B_T/h*)·exp(−Δ*G*^≠^/*RT*) (1)

where *k*_B_ is the Boltzmann constant, *h* is the Planck constant, *T* is the reaction temperature, and *R* is the universal gas constant. Since this paper focuses on the support effect and the relative free energy for two similar reactions can be more reliable than the absolute free energy profile for one reaction with the state-of-the-art DFT calculation, the relative rate constant of the supported case over that of the unsupported case was calculated in this work. From Equation (1), the following equation can be easily derived:

*k*_BN-sup_/*k*_unsup_*=* exp[(Δ*G*_unsup_^≠^ − Δ*G*_BN-sup_^≠^)/*RT*] (2)

In Equation (2), the left side term is the relative rate constant, and the subscripts “BN-sup” and “unsup” stand for the reactions involving and not involving the model support, respectively. Δ*G*^≠^ can be calculated from the relative free energy of **TS** over **IM1** for all the reactions of (R1)~(R6). We calculated the relative rate constants at two temperatures of 298.15 K and 673.15 K, since the former is widely concerned in general physical chemistry [35], and the latter is a typical temperature for Ru-catalyzed NH_3_ decomposition to H_2_ in practice [4,12,15,24]. By collecting the free energy data at 298.15 and 673.15 K (Table 2) for all of the **TS**s and **IM1**s in this work, and based on Equation (2), the relative rate constants, *k*_BN-sup_/*k*_unsup_, can be easily calculated as 20.1, 2.8, and 3.2 for Ru_1_, Ru_2_, and Ru_3_ clusters, respectively, at 298.15 K. These *k*_BN-sup_/*k*_unsup_ values are 775, 9.4, and 272 for Ru_1_, Ru_2_, and Ru_3_ cases, respectively, at 673.15 K. The calculated data indicate that the involvement of the model BN support leads to a great influence on the reaction rate constant for N-H bond activation, especially for the single atom Ru catalyst, and at high reaction temperatures.

Our previous works studied the silica support effect on the C-H bond activation of ethane on a nickel oxide cluster [36]. In that work, the energy of activation of C-H bond activation of ethane on a nickel oxide cluster is increased (instead of decrease in this paper) by the involvement of the silica support. Cao et al. studied the support effect on the Pd-catalyzed semi-hydrogenation of acetylene from the structural and kinetic perspectives [37]. They found that, compared with Al_2_O_3_, the CNT support reduced the Pd^0^ 3d binding energy and suppressed the formation of PdH_x_ species to enhance the reaction kinetics in terms of the ethylene selectivity and formation rate. These different effects on the energy of activation (consequently the reaction rate constant) may be caused by the difference of the support nature, and the computational study like this work can help researchers in the rational selection of good catalytic supports.

The existing literature, exemplified by a review article of ref. [38], clearly indicates that the catalytic support significantly influences the efficiency of the NH_3_ decomposition for hydrogen production. Compared to traditional oxide supports (such as alumina and magnesium oxide), Ru catalysts supported on novel carbon materials (e.g., graphene) exhibit a markedly superior performance [39,40]. Due to the complexity of the ammonia decomposition reaction mechanism, it remains unclear how supports accelerate the entire decomposition process. Nevertheless, our research results suggest that the inclusion of a carbon analogue support, h-BN, can accelerate the first (and certainly crucial) step of the N-H bond activation (see *k*_BN-sup_/*k*_unsup_ values shown above). The theoretically predicted trends of acceleration are qualitatively aligned with the trends observed in ammonia decomposition reactions involving graphene (h-BN analogue) as the catalytic support.

#### 2.5.3. Cluster Size Effect

Combining the findings described in Section 2.5.1 and Section 2.5.2, one can have a different view angle of the cluster size effect. For the unsupported cases, with using the **SM** as the energetic reference, the stability of either **IM1** or **IM2** increases with the order of Ru_1_ < Ru_2_ < Ru_3_. The involvement of BN makes the stability order for the IM1 case changes to the order of Ru_1_ > Ru_2_ > Ru_3_, with the stability of **IM2** still maintaining the order of Ru_1_ < Ru_2_ < Ru_3_.

In the kinetic aspect, the free energy of activation follows an increasing order of Ru_2_ < Ru_1_ < Ru_3_, for both of the unsupported and supported cases, and thus the theoretical rate constant follows the decreasing order of Ru_2_ > Ru_1_ > Ru_3_. However, the degree of influence on the reaction rate constant induced by the BN support follows the order of Ru_1_ > Ru_3_ > Ru_2_.

#### 2.5.4. Support Effect on the Electron Transfer from IM1 to TS

Table 3 shows the NBO charge changes of different moieties for the process of **IM1** going to **TS**. During the **IM1 → TS** process, Ru_n_ clusters undergo electron loss, while NH_2_ fragments containing hydrogen atoms (H_a_) experience electron gain.

During the transformation from **IM1** to **TS** in all six cases in this work, there is a loss of charge in the Ru atom/cluster. For the Ru_1_ system, upon introducing the BN support, the charge lost amount of the Ru atoms during the **IM1→TS** transformation noticeably decreases (from 0.418 to 0.086, see Table 3). Simultaneously, the BN support loses 0.159 of its charge. It is not difficult to speculate that the BN support shares a portion of the charge loss with the Ru atoms. For the Ru_2_ and Ru_3_ cases, upon introducing the support, the charge lost amount of the Ru atoms during the **IM1→TS** process slightly increases. Table 3 also indicates that the BN support has only a slight charge change (Ru_2_-BN, 0.025; Ru_3_-BN, −0.047). It no longer directly assists in the charge transfer of Ru atoms. A possible reason is that the distance between the activation sites in Ru_2_/Ru_3_ clusters and the BN support increases compared to the Ru_1_ case.

During the **IM1→TS** process, the H_a_ atom gains electron for all six cases. The involvement of the BN support leads to a decrease in the electron gain for the H_a_ atom. At the same time, as can be seen in Table 2, the involvement of the BN support leads to a decrease in N-H bond activation free energy. Putting the results of NBO analysis and the reaction energy barrier together, one can further find that the trend in the electron transfer of H_a_ is consistent with the change of reaction energy barrier. The decrease in the reaction barrier introduced by the BN support can be associated with its electron transfer behavior.

#### 2.5.5. Support Effect on the Spin Conversion Behavior for the MFPs

Table 4 shows the spin multiplicity of intermediates and transition states in the MFP on the **Ru_n_-unsup** and **Ru_n_-BN** clusters. The main changes for the spin states introduced by the incorporation of the BN support are as follows. Firstly, Table 4 shows that the ground states of all **SM** conform to a regular pattern, with the most favorable spin multiplicity being 2*n* + 3 where “*n*” is the number of Ru atoms. The involving BN support does not change the ground states of the metal clusters. Second, the changes of the spin multiplicity of the intermediate and transition state in the MFP by the BN support is shown by the fact that the most favorable spin multiplicity is not changed for the *n* = 1 and 3 cases, and the most favorable spin multiplicity is changed for the *n* = 2 case. No spin transition occurs at the Ru_3_ cases. Finally, the involving BN support changes the spin multiplicity of **IM1** and **IM2** at *n* = 2, from **^7^IM1** to **^5^IM1** and from **^7^IM2** to **^7^IM2**.

The current literature shows that the reaction of NH_3_ decomposition to generate H_2_ at low temperatures is unsatisfactory. Based on the data obtained, it is reasonable to speculate that the reaction requires high temperatures to induce the transition of the spin states in the intermediates and transition states. When the energies of the two spin states are close, various external perturbations like temperature, pressure, and magnetic field can induce the spin state transition or crossover [41]. The spin state of the transition metal affects the magnetization strength and thermal conductivity of the material to change the thermoelectric properties of the material [42]. Inspection of the spin state data in Table 4 data shows that the state crossover occurs in most cases. So, it is reasonable to speculate that a suitable support may help improve the N-H bond activation rate during NH_3_ decomposition to generate H_2_ at low temperatures. In the long term, with the aid of computational tools, the findings in this paper will provide a promising direction for designing a good catalyst for H_2_ generation from NH_3_ decomposition at low temperatures.

#### 2.5.6. Preliminary Orbital Analysis

**Figure 9:**
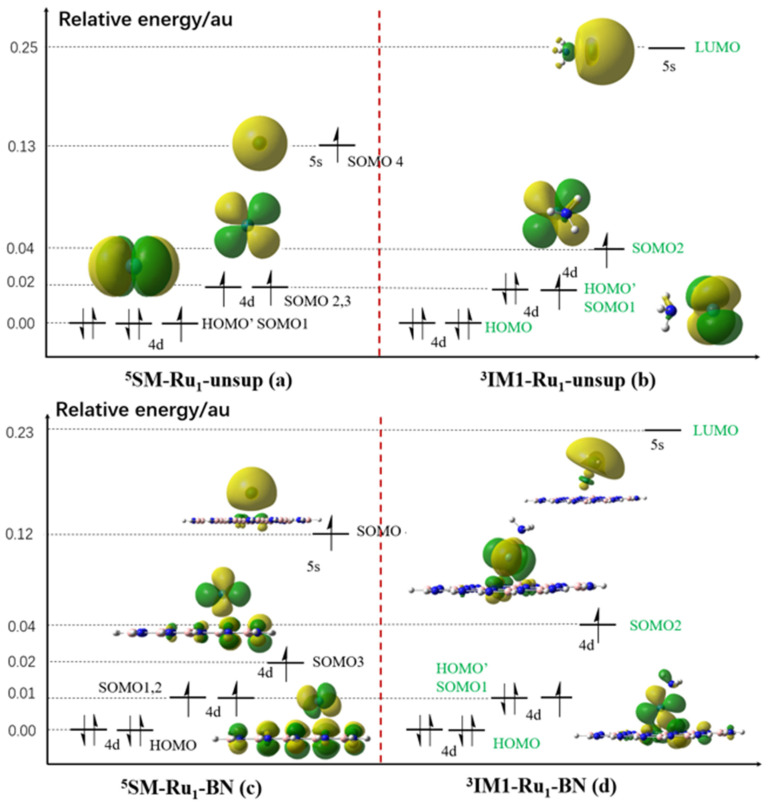
The molecular orbital contours and the relative orbital energy (in atomic unit, a.u.) of some selected frontier orbitals (see the text for detail) of ^5^**SM-Ru1-unsup** (**a**), ^3^**IM-Ru1-unsup** (**b**), ^5^**SM-Ru1-BN** (**c**), and ^3^**IM-Ru1-BN** (**d**). For the relative orbital energies, the HOMO energy is selected as the energetic reference (0 atomic unit, a.u.). Since NH_3_ is a singlet species, the orbital image of NH_3_ is not shown for the **SM**s.

In principle, the ground spin multiplicity of a certain species can be explained by the relative energy of the frontier orbitals of this species, namely the highest occupied molecular orbital (HOMO), the singly occupied molecular orbital(s) (SOMO), and the lowest unoccupied molecular orbital (LUMO). In order to better understand the spin transition behavior of some key species involved in the N-H bond activation processes in this work, in the preliminary stage we focused on understanding the ground spin multiplicity of ^5^**SM-Ru1-unsup**, ^3^**IM-Ru1-unsup**, ^5^**SM-Ru1-BN,** and ^3^**IM-Ru1-BN**. Figure 9 shows the relative energy/energy split of the HOMO, SOMO, and LUMO of these four species as well as their orbital contours. Since NH_3_ is a singlet species, the orbital image of NH_3_ is not shown for the **SM**s. It is well known that an Ru atom has a quintet state, that is, having four singly occupied electrons. Figure 9a shows SOMO-1~3 orbitals of ^5^**SM-Ru1-unsup** (actually an Ru atom) having Ru 4d characters are nearly degenerated, and SOMO-4 having Ru 5s character. The energy split between this Ru 5s orbital and Ru 4d in SOMO-3 is 0.11 atomic unit (a.u.). Figure 9b shows the approaching of NH_3_ to Ru to form **^3^IM-Ru1-unsup,** making the energy split of this Ru 5s orbital over the Ru 4d orbital significantly increased (energy split of 0.21 a.u), and thus the electron in SOMO-4 in ^5^**SM-Ru1-unsup** tend to occupy SOMO-1 to form an electron pair. Therefore, SOMO-1 in ^5^**SM-Ru1-unsup** changes to a new HOMO′ in **^3^IM-Ru1-unsup**, making it having a triplet ground state.

Figure 9c shows that when a BN support approaches the Ru atom to form **SM-Ru_1_-BN**, the energy split of the Ru 5s orbital with the Ru 4d orbital in SOMO-3 is only 0.10 a.u., making SOMO-4 still being occupied by an electron in **SM-Ru_1_-BN**, thus having a quintet state. A similar reason for why **IM1-Ru_1_-BN** having a triplet ground state can be found compared to the case of **IM1-Ru1-unsup** according to the energy values shown in Figure 9d.

## 3. Computational Methods and Reactant Models

The DFT calculations were performed by employing the M062X [43] exchange and correlation functionals to explore the potential energy surfaces (PESs) of the first N-H bond activation process of NH_3_. To better describe the long-term interaction between NH_3_ and Ru or BN nanosheet due to a dispersion problem, the Grimme’s D3 dispersion correction [44] was applied for all DFT calculations. The activation process operates on the Ru_n_ (*n* = 1, 2, 3) clusters without and with B_19_N_19_H_16_ as the model BN support [33]. This model support is denoted as “-BN” appearing in a certain species notation hereafter in this paper. M062X is known to be able to provide a good description of the PES for the bond activation process on transition metal clusters [34,45] as well as for the BN-involved reaction system [46]. In the present status, although DFT cannot easily provide a quantitative explanation of the experimental data, the relative reaction barrier is much more credible [47]. The basis information set will be specified after the description of the model of reactants. All PESs were explored by optimizing the geometries in the energy minimums for the reactants, the intermediates, and the products, and the first-order saddle points for transition states using the Gaussian 09 program suite (B.09 (for initial optimization) and C.01 (for final optimization and frequency analysis) versions [48,49]). Frequency analyses were performed to confirm the energy minimums and the first-order saddle points, as well as to obtain the zero-point corrected energies of the optimized geometries. Intrinsic reaction coordinate (IRC) computations [50] were performed to confirm the transition states connecting the appropriate reactants and products.

Since this paper emphasizes an understanding of the support effect of a model BN on the N-H bond activation, we investigated and compared the structural and energetic data for the interaction of supported and unsupported Ru metal clusters with one NH_3_ molecule. In order to directly understand the role of model BN support, all of the 6 reactions interested in this paper are categorized into two types, and expressed as follows.

The first type corresponds to the unsupported cases, i.e., the reaction of NH_3_ with an unsupported Ru_n_ cluster (where *n* = 1, 2, or 3) to form the NH_2_-Ru_n_-H species, which includes the following three reactions:

NH_3_ + Ru_1_ → NH_2_-Ru_1_-H (R1)

NH_3_ + Ru_2_ → NH_2_-Ru_2_-H (R2)

NH_3_ + Ru_3_ → NH_2_-Ru_3_-H (R3)

The second type corresponds to the supported cases, i.e., the reaction between NH_3_ and model BN-supported Ru_n_ cluster (denoted as Ru_n_-BN, where *n* = 1, 2, 3) to afford NH_2_-Ru_n_-H-BN species, which includes the following three reactions in detail:

NH_3_ + Ru_1_-BN → NH_2_-Ru_1_-H-BN (R4)

NH_3_ + Ru_2_-BN → NH_2_-Ru_2_-H-BN (R5)

NH_3_ + Ru_3_-BN → NH_2_-Ru_3_-H-BN (R6)

For the above 6 reactions, the key species on different PESs with a certain spin multiplicity (*S*) were optimized in geometries and energetically calculated. For easy description hereafter in this paper, the notations for different key species on different PESs are defined as in the following regulations.

Firstly, these key species include the starting materials (**SM**), the first intermediate formed from the **SM**, **IM1**, the transition state followed by **IM1**, **TS**, and the second intermediate followed by **TS**, **IM2**. The **SM** is actually the system of separated NH_3_ and one of the six Ru clusters in the left side of (R1)~(R6), and **IM2** is actually the first N-H bond activation product of one of (R1)~(R6). Secondly, since all species in all of the six reactions have an even number of electrons, the PESs with different multiplicities of *S* = 1, 3, 5, 7… (i.e., singlet, triplet, quintet, heptet, and so on) were explored. The information about *S* is put in the upper-left superscript in front of a species notation to indicate its spin multiplicity. For example, ^7^Ru_3_ is a heptet Ru_3_ cluster, and **^3^TS** is a triplet transition state. Therefore, possibly the most complicated notation for a certain species in this paper can be expressed as *^S^***(SM, IM1, TS, or IM2)-Ru_n_-(unsup or BN)**, where the suffix “-unsup” stands for the unsupported case, and “-BN” stands for the model BN-supported cases. For example, **^7^IM2-Ru_3_-BN** means the **IM2** from the reaction of NH_3_ with a model BN-supported Ru_3_ cluster with a heptet state, and **^3^TS-Ru_2_-unsup** means the **TS** in the reaction of NH_3_ with an unsupported Ru_2_ cluster with a triplet state under the above name regulation.

**Figure 10:**
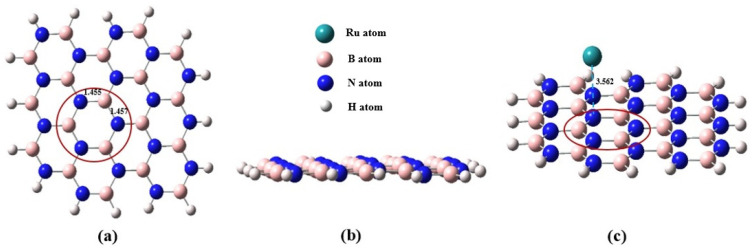
Vertical (**a**) and side (**b**) views of the model BN of a B_19_N_19_H_16_ sheet, and the **^5^**Ru_1_-BN model (**c**). The Ru atom is located close to the indicated N atom with a distance of 3.562 Å. For the B and N atom in the cycle, a high level basis set of 6-311G** was used for calculation as described in the text. Color for each atoms: N (blue), H (white), Ru (green), and B (pink); see the inset over panel (**b**).

Figure 10 shows the M06X-GD3 optimized geometries of the B_19_N_19_H_16_ sheet and ^5^Ru_1_-BN (c) in different views. For B_19_N_19_H_16_, the 6-311G** basis set was used for atoms in the red circles as indicated in Figure 10a,c. The 6-31G basis set was used for the residual atoms of B_19_N_19_H_16_. Two levels of basis sets were used for describing the model BN support in order to compromise between the computational accuracy and the time expense. The SDD basis set was used for the Ru atoms [51]. The 6-311G** basis set was also used for N and H atoms in NH_3_. The cluster model was used for the calculations in this work. For all of the structure optimization and energy calculations, all of the atoms were allowed to relax. Hereafter in this paper, for all the geometries in the supported cases, only the side view of the BN support will be shown in Section 2 unless specially specified.

## 4. Conclusions

To gain deeper insight into the influence of a BN support on the N-H bond activation reaction, the optimized geometries and energetics calculated with the DFT method for the first N-H bond activation of NH_3_ on unsupported Ru_n_ clusters and on Ru_n_-BN clusters were compared. This DFT study provides the following primary conclusions:

(1) From a geometric standpoint, the incorporation of the BN support does not lead to obvious alterations of the structure of the intermediates and transition states involved in the most favorable pathway (MFP). This is mainly reflected by slight changes in the distance between the H_a_ and N atoms in NH_3_ in the **TS**s, **IM1**s, and **IM2**s when the unsupported and BN-supported cases are compared. 

(2) Considering thermodynamics, the formation of **IM2** is favored by the presence of the BN support for all Ru_n_ clusters. In contrast, the formation of **IM1** is favored for the Ru_1_ and Ru_2_ cases, and disfavored for the Ru_3_ case by the presence of BN.

(3) In terms of kinetics, the incorporation of the BN support leads to a decrease in the free energy of activation of the first N-H bond activation process of NH_3_, and thus can improve the reaction rate constant. The rate constant improvement induced by the BN support is more significant at high temperatures.

(4) Spin transition occurs in the MFP in (R1), (R2), (R4) and (R5) for the Ru_1_ and Ru_2_ cases, and no spin transition occurs in the MFP in (R3) and (R6) for the Ru_3_ cases. The incorporation of the BN support changes the spin transition behavior for the Ru_2_ cluster during the first N-H bond activation of NH_3_.

The spin transition behavior connecting to the single and gemini-Ru atom catalysts underscores the importance of considering spin transition behavior when choosing catalytic supports, particularly in the field of single atom catalysis.

Our study contributes to a deeper understanding of the N-H bond activation process in catalytic NH_3_ decomposition. These insights offer valuable guidance for selecting more favorable catalytic supports in order to synthesize better catalysts. We will continue to carry out further works to provide better theoretical guidance for the design of efficient catalysts for H_2_ production via NH_3_ decomposition.

## Figures and Tables

**Table 1:** Adsorption energy (*E*_ad_) and free energy at 298 K (*G*_ad_) of an Ru_n_ cluster on the BN support. All the spin multiplicity indicated on the upper left of a species notation corresponds to the ground spin state.

Adsorption Process	*E*_ad_ (kcal/mol)	*G*_ad_ (kcal/mol, 298 K)
^5^Ru_1_ + ^1^BN → ^5^Ru_1_-BN	−11.7	−4.4
^7^Ru_2_ + ^1^BN → ^7^Ru_2_-BN	−29.0	−6.4
^9^Ru_3_ + ^1^BN → ^9^Ru_3_-BN	−32.2	−10.6

**Table 2:** Free energy of activations, Δ*G*^≠^ (in kcal/mol), at 298.15 and 673.15 K for all the six N-H bond activation reactions (R1)~(R6) studied in this work.

N-H Activation of NH_3_ on	Unsupported, 298.15 K	BN-Supported, 298.15 K	Unsupported, 673.15 K	BN-Supported, 673.15 K
Ru_1_ atom	26.0	24.1	25.8	16.9
Ru_2_ cluster	21.8	21.1	24.0	21.0
Ru_3_ cluster	29.8	28.9	32.5	25.0

**Table 3:** NBO charge change of different moieties between the most favorable **IM1** and **TS** involved in the first N-H bond activation process of NH_3_ on **Ru_n_-unsup** and **Ru_n_-BN** clusters **(IM1→TS)**.

	NBO Charge Change of Different Moieties				
	H_a_	N	NH_2_	Ru_n_	B_19_N_19_H_16_
Ru_1_-unsup	−0.214	−2.207	−2.204	0.418	0.000
Ru_1_-BN	−0.190	0.021	−0.055	0.086	0.159
Ru_2_-unsup	−0.283	−0.037	−0.056	0.339	0.000
Ru_2_-BN	−0.251	−0.098	−0.146	0.372	0.025
Ru_3_-unsup	−0.274	−0.024	−0.058	0.332	0.000
Ru_3_-BN	−0.240	−0.087	−0.130	0.417	−0.047

**Table 4:** Spin multiplicity of intermediates and transition states in most favorable pathways for the first N-H bond activation of NH_3_ with and without the BN support.

	Most Favorable Spin State			
	SM	IM1	TS	IM2
Ru_1_-unsup	5	3	3	3
Ru_1_-BN	5	3	3	3
Ru_2_-unsup	7	7	5	7
Ru_2-_BN	7	5	5	5
Ru_3_-unsup	9	9	9	9
Ru_3_-BN	9	9	9	9

## Data Availability

All data are available upon request.
